# Etymologia: *Edwardsiella tarda*

**DOI:** 10.3201/eid2510.ET2510

**Published:** 2019-10

**Authors:** Ronnie Henry

**Keywords:** *Edwardsiella tarda*, bacteria, Philip R. Edwards, facultatively anaerobic, opportunistic pathogens, fish, amphibians, reptiles, zoonoses

## *Edwardsiella tarda* [ed-wahrdʺse-elʹǝ tarʹdǝ]

In 1965, a group of CDC researchers described a species of gram-negative, facultatively anaerobic bacteria in the family *Enterobacteriaceae*, which they named *Edwardsiella* (for CDC microbiologist Philip R. Edwards) *tarda* (Latin, “slow,” referring to biochemical inactivity and the fact that it ferments few carbohydrates) (Figure). These organisms infect a variety of fish, reptiles, and amphibians and are opportunistic pathogens for humans.

**Figure:**
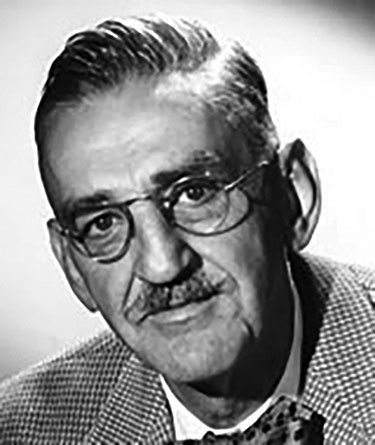
Dr. P.R. Edwards of the US Public Health Service seated in the background, and George Herman working in the Enteric Bacteriology Unit Laboratory. Dr. Edwards joined the staff of the Communicable Disease Center of the Public Health Service in 1948 and served as Chief of the Enteric Bacteriology Unit until June 1962, when he accepted the post of Chief of the Bacteriology Section at CDC. Image source: Public Health Image Library.
